# Reference genes for quantitative real-time PCR analysis in symbiont *Entomomyces delphacidicola* of *Nilaparvata lugens* (Stål)

**DOI:** 10.1038/srep42206

**Published:** 2017-02-13

**Authors:** Pin-Jun Wan, Yao-Hua Tang, San-Yue Yuan, Jia-Chun He, Wei-Xia Wang, Feng-Xiang Lai, Qiang Fu

**Affiliations:** 1 State Key Laboratory of Rice Biology, China National Rice Research Institute, Hangzhou 310006, China

## Abstract

*Nilaparvata lugens* (Stål) (Hemiptera: Delphacidae) is a major rice pest that harbors an endosymbiont ascomycete fungus, *Entomomyces delphacidicola* str. NLU (also known as yeast-like symbiont, YLS). Driving by demand of novel population management tactics (e.g. RNAi), the importance of YLS has been studied and revealed, which greatly boosts the interest of molecular level studies related to YLS. The current study focuses on reference genes for RT-qPCR studies related to YLS. Eight previously unreported YLS genes were cloned, and their expressions were evaluated for *N. lugens* samples of different developmental stages and sexes, and under different nutritional conditions and temperatures. Expression stabilities were analyzed by BestKeeper, geNorm, NormFinder, ΔCt method and RefFinder. Furthermore, the selected reference genes for RT-qPCR of YLS genes were validated using targeted YLS genes that respond to different nutritional conditions (amino acid deprivation) and RNAi. The results suggest that *ylsRPS15p*/*ylsACT* are the most suitable reference genes for temporal gene expression profiling, while *ylsTUB/ylsACT* and *ylsRPS15e*/*ylsGADPH* are the most suitable reference gene choices for evaluating nutrition and temperature effects. Validation studies demonstrated the advantage of using endogenous YLS reference genes for YLS studies.

Reverse transcription quantitative polymerase chain reaction (RT-qPCR) is the most popular and indispensable tool in molecular biology^1^,^2^. RT-qPCR assay allows fast, accurate and sensitive measurement of target mRNA during the exponential amplification phase by detecting fluorescent markers directly related to the amount of amplified target. The technique combines amplification, detection and quantification in a single step. RT-qPCR assay measures cycle threshold (Ct, also referred as quantification cycle or crossing point that is abbreviated as Cq or Cp), defined as the PCR cycles at which the fluorescent signal of the reporter dye crosses an arbitrarily set threshold. One direct and precise quantification method is using standard curves in which samples of known quantity are serially diluted and then amplified. The generated standard curve can then be used to quantify samples of unknown quantity. However, the most popular tool is comparative RT-qPCR, where the quantification is achieved through Ct difference (ΔCt) between the gene of interest and a housekeeping gene(s) (also named as reference gene or normalizing gene) in the same sample without knowing the absolute level of expression^3^.

The ΔCt method requires the chosen reference gene(s) to have an almost constant level of expression across all conditions/samples, because Ct values can be influenced by variations in RNA extraction (RNA quality and quantity), reverse transcription, cDNA concentration, and PCR reaction (including components, parameters and reaction efficiency)^4^. To obtain reliable and valid gene expression profiles, stable internal controls are critical for normalizing expression^1^. Reference genes are mostly selected from housekeeping genes (HKGs) because they are essential to the basic cellular survival and normally are expressed constitutively in all cells regardless of conditions (tolerant to regulations)^1^,^2^,^5^,^6^,^7^,^8^. A variety of HKGs have been evaluated and utilized as reference genes in different organisms^5^,^9^,^10^. For examples, *γ-globin*, *NADH oxidase* (*NOX*), *aldolase* (*ALD*), *hydroxymethylbilane synthase*, *hypoxanthine phosphoribosyl transferase 1*, *β-actin* (*ACTB*), *glyceraldehyde-3-phosphate dehydrogenase* (*GAPDH*) and *18S ribosomal RNA* (*18S rRNA*) have been validated and used extensively as reference genes for expression studies of many different organisms^10^,^11^,^12^,^13^,^14^, and other genes (e.g. *ubiquitin 10* and *tubulin 6*, that were abbreviated as *UBQ10* and *TUB6*, respectively) have been ruled unsuitable as references^15^. According to the Minimum Information for Publication of Quantitative Real-Time PCR Experiments (MIQE) guidelines^16^, HKGs are now selected based on their specificity in interactions between a species or cell type/s subjected to different treatments or conditions. This recommendation requires more detailed expression characterization of potential HKGs in various samples and conditions. More recent, the increasing popularity of the high-throughput next generation sequencing technologies (HT-NGS) has led to a significant increase in transcriptomic and genomic resources of various organisms^17^,^18^,^19^,^20^. RT-qPCR is one powerful and widely used tool to characterize and assess the quality of these genetic data^1^,^2^, making studies of potential reference gene characteristics more urgent.

The brown planthopper, *Nilaparvata lugens* (Stål) (Hemiptera: Delphacidae), is a major pest of rice in Asia^21^,^22^,^23^,^24^. Novel population management tactics are in high demand. *N. lugens* harbors an endosymbiont ascomycete fungus, *Entomomyces delphacidicola* str. NLU (also known as yeast-like symbiont, YLS) that has been revealed to play an important role in amino acid biosynthesis for *N. lugens*^25^. In recent years, new technologies such as NGS and RNA interference (RNAi) offer the possibility of understanding the molecular principles of *N. lugens* and YLS interaction, and provide potential opportunities for novel pest management^24^,^26^,^27^ as high mortality can be resulted from double-stranded RNA (dsRNA)-mediated knockdown of *N. lugens* originated genes^28^,^29^,^30^,^31^,^32^,^33^. Similarly, dsRNA knockdowns of YLS originated genes offer another valid population management option that can provide extra safety for non-target organisms with no YLS^34^. Our laboratory has identified YLS originated genes involved in essential amino acid biosynthesis pathways for *N. lugens* that could potentially serve as candidates for RNAi^34^,^35^. Although *N. lugens* originated housekeeping genes including *ribosomal protein S11e* (*NlRPS11*), *NlRPS15*, *α2-tubulin* (*NlTUB*) and *β-actin* (*NlACT*) have been used as reference genes for YLS studies^34^, using endogenous YLS genes would be more convincible for accurate expression profiling.

In this study, we identified and cloned eight previously unreported YLS genes as reference gene candidates. The expression stabilities of these genes were evaluated for *N. lugens* samples of difference developmental stages and sexes, and under different temperatures and nutritional conditions. Furthermore, the suitability of these genes as reference controls for RT-qPCR assays of YLS genes that respond to different nutritional conditions (amino acid deprivation) and RNAi were evaluated.

## Results

### Expression of YLS housekeeping gene candidates

Using the pooled insect samples, the expression of the eight selected HKGs of YLS was investigated by RT-qPCR. All genes were expressed and visualized as a single band of the expected sizes on 2% agarose gels. All amplicons were sequenced and displayed 99–100% identity with the corresponding gene sequences. The PCR efficiency (calculated from the standard curve) and correlation coefficient (R^2^) are shown in Table 1. The primer efficiency ranges between 93% and 105% with R^2^ of 0.995–1.000. These data indicate that amplification efficiencies of the primers met the standard requirement of conventional RT-qPCR (efficiency between 90% and 110% with R^2^ of 0.985–1.000).

**Figure 1:**
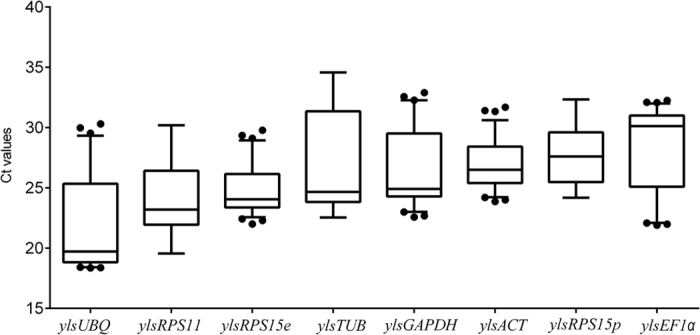
Expression profiles of candidate reference genes in *N. lugens*. The expression level of candidate references in all tested samples are documented in Ct-value, and showed as boxplot. The dot indicates the maximum or minimum value of Ct, while whiskers indicate the standard error (SE) of the mean.

The raw Ct values of all tested samples reveal that these genes were expressed at different levels (average Ct between 19.72 and 30.12) with different levels of variation (standard error, SE between 0.2350 and 0.5021). As shown in Fig. 1, *ylsEF1α* expressed at the lowest level (the highest Ct value), followed by *ylsRPS15p*, *ylsACT*, *ylsGAPDH*, *ylsTUB*, *ylsRPS15e*, *ylsPRS11* and *ylsUBQ.* Among them, *YlsTUB*, *ylsUBQ* and *ylsEF1α* had higher expression variation, whereas the other five genes had lower expression variation (*ylsACT* < *ylsRPS15e* < *ylsRPS15p* < *ylsGAPDH < ylsPRS11*). The detection of gene specific expression variation of different samples signifies the importance of customizing reference gene selection according to sample types and experimental conditions.

### Stability of reference gene candidates

To identify the most stable reference gene of the different sample types, the expression stabilities were evaluated by the ΔCt method, NormFinder, geNorm and BestKeeper, respectively. The overall stability ranking was obtained by RefFinder.

### Development and sex

**Figure 2:**
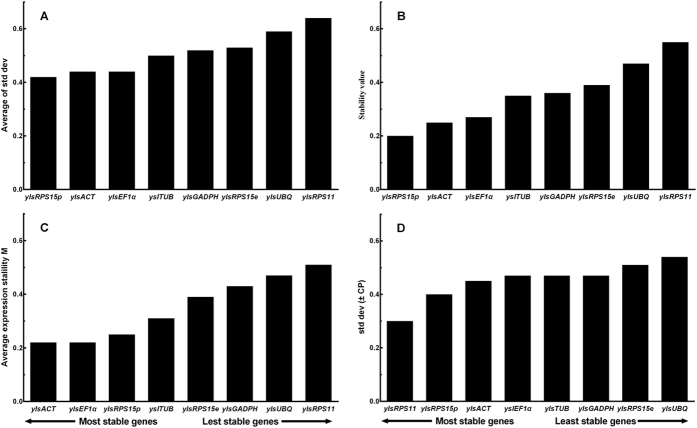
Stability of the reference gene expression in different developmental stages of *N. lugens*. Candidate genes (*ylsActin*, *ylsEF1α*, *ylsGADPH*, *ylsRPS11*, *ylsRPS15e*, *ylsRPS15p*, *ylsTub*, *ylsUBQ*) were evaluated using the comparative ΔCt method (**A**), NormFinder (**B**), geNorm (**C**) and BestKeeper (**D**).

**Figure 3:**
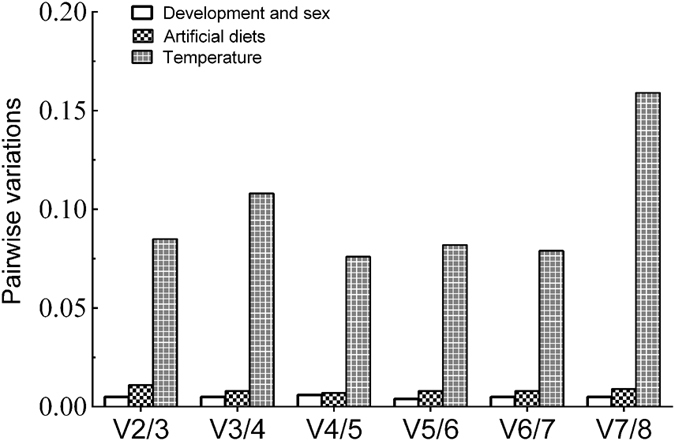
Optimal number of reference genes for normalization in *N. lugens*. The pairwise variation (Vn/Vn+1) was analyzed by the geNorm software to determine the optimal number of reference genes for RT-qPCR. The average values of pairwise variation (V) dictate whether inclusion of an extra reference gene would add to the stability of the normalization factor.

The ΔCt method indicated that *ylsRPS15p*, *ylsACT* and *ylsEF1α* were the most stable reference genes across samples of different developmental stages and sexes (Fig. 2A). The lower expression stability values showed by NormFinder also indicated that *ylsRPS15p*, *ylsACT* and *ylsEF1α* were the most stably expressed genes (Fig. 2B). The estimation of geNorm shows that *ylsACT*, *ylsEF1α* and *ylsRPS15p* were the most stable genes, and *ylsPRS11* and *ylsUBQ* were the most unstable genes (Fig. 2C). Analysis using BestKeeper indicated that *ylsACT*, *ylsEF1α, ylsRPS15p* and *ylsTUB* were the most stable reference genes (Fig. 2D). RefFinder ranks the genes from most to least stable as the following: *ylsRPS15p* > *ylsACT > ylsEF1α > ylsTUB* > *ylsPRS11 > ylsGAPDH* > *ylsRPS15e* > *ylsUBQ* (Table 2). Furthermore, geNorm analysis revealed that all pairwise variations (Vn/Vn + 1) were below 0.15 (the recommended threshold of cut-off), indicating that the inclusion of additional reference is unnecessary (Fig. 3). Thus, the best combination of reference genes for developmental stage and sex samples of *N. lugens* are *ylsRPS15p* and *ylsACT*.

### Different nutritional conditions

**Figure 4:**
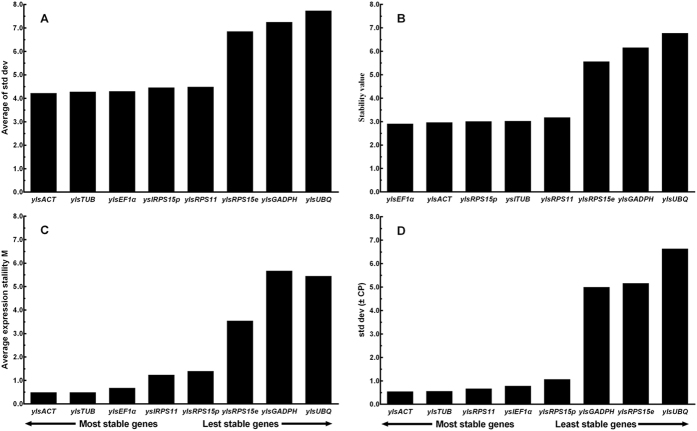
Stability of the reference gene expression in *N. lugens* reared on artificial diets that missed one of the 20 amino acids. Candidate genes (*ylsActin*, *ylsEF1α*, *ylsGADPH*, *ylsRPS11*, *ylsRPS15e*, *ylsRPS15p*, *ylsTub*, *ylsUBQ*) were evaluated using the comparative ΔCt method (**A**), NormFinder (**B**), geNorm (**C**) and BestKeeper (**D**).

Analyses of ΔCt method, NormFinder and BestKeeper divided the genes into two groups: more stably expressed *ylsACT*, *ylsTUB*, *ylsEF1α*, *ylsRPS15p* and *ylsPRS11*; and less stably expressed *ylsUBQ, ylsRPS15e* and *ylsGAPDH* (Fig. 4A,B,D). The geNorm analysis divided the genes into three groups: the most stably expressed *ylsACT*, *ylsTUB* and *ylsEF1α* (M < 1); the least stable expressed *ylsUBQ, ylsRPS15e* and *ylsGAPDH* (M > 3); and a middle group of *ylsPRS11* and *ylsRPS15p* (1 < M < 3) (Fig. 4C). RefFinder ranking from most to least stable is *ylsACT* > *ylsTUB* > *ylsEF1α > ylsRPS15e* > *ylsGAPDH* > *ylsPRS11 > ylsRPS15p* > *ylsUBQ* (Table 2). The geNorm analysis also revealed that all pairwise variation (V_n_/V_n + 1_) values were below 0.15 (Fig. 3). Thus, the combination of reference genes recommended for this sample type is *ylsTUB* and *ylsACT*.

### Temperature conditions

**Figure 5:**
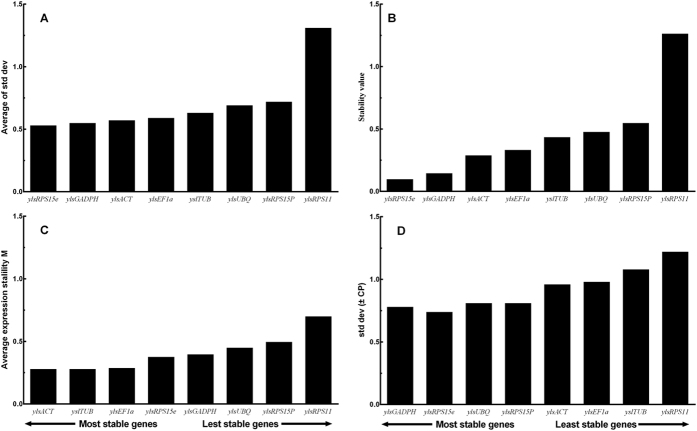
Stability of the reference gene expression in *N. lugens* reared on rice under different temperatures. Candidate genes (*ylsActin*, *ylsEF1α*, *ylsGADPH*, *ylsRPS11*, *ylsRPS15e*, *ylsRPS15p*, *ylsTub*, *ylsUBQ*) were evaluated using the comparative ΔCt method (**A**), NormFinder (**B**), geNorm (**C**) and BestKeeper (**D**).

With the samples exposed to different temperature conditions, the comparative ΔCt method, NormFinder and BestKeeper analyses indicated that *ylsRPS15e* and *ylsGAPDH* were the most stable genes, while *ylsACT* and *ylsTUB* were the most stable genes found by geNorm (Fig. 5). According to RefFinder, the overall order from most to least stable is *ylsRPS15e* > *ylsGADPH* > *ylsACT* > *ylsTUB* > *ylsEF1α* > *ylsUBQ* > *ylsRPS15p* > *ylsRPS11* (Table 2). The geNorm analysis revealed that almost of all pairwise (V_n_/V_n + 1_) variations were below 0.15 (Fig. 3). Thus, the combination of reference genes recommended for this subset is *ylsRPS15e* and *ylsGADPH*.

### Validation of reference gene selection

**Figure 6:**
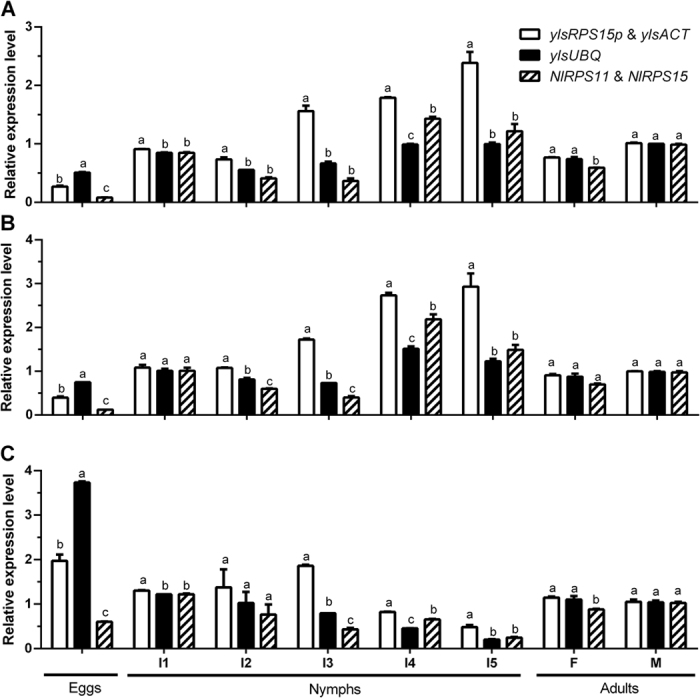
Validation of reference gene selection in samples of developmental stages and sex. The expression of target genes *ylsSDH* (**A**), *EdPRTase* (**B**) and *ylsArg4* (**C**) was determined in eggs, nymphs (the first to fifth instar, I1 to I5), female and male adults (F and M), respectively. The data are denoted as mean ± SE. Different letters above the bars represent significant differences among samples of developmental stages and sexes using one of the three reference gene types (*P* = 0.05) (Turkey’s test).

Recently, the temporal expression patterns of *saccharopine dehydrogenase* (*NlylsSDH*) and *ATP-phosphoribosyltransferase* (*EdePRTase*) were determined by using two *N. lugens* reference genes *NlRPS11* and *NlRPS15* as internal controls^34^,^36^. In the current study, the relative expression of *NlylsSDH*, *EdePRTase* and *argininosuccinate lyase* (*ylsArg4*) in *N. lugens* samples of different developmental stages and sexes was determined using the YLS reference gene pair *ylsRPS15p* and *ylsACT*, an identified unstable gene *ylsUBQ* and the *N. lugens* reference gene pair *NlRPS11* and *NlRPS15*. Two-way ANOVA was performed to evaluate the effect of the reference genes and the developmental stages. As expected, the expression profiles of the two target genes (*NlylsSDH* and *EdePRTase*) showed significant difference among developmental stages, but the expression profile of *ylsArg4* was not significant different at *P* = 0.05 (*NlylsSDH*: F_7, 24_ = 4.938, *P* = 0.0054; *EdePRTase*: F_7, 24_ = 6.174, *P* = 0.0019; *ylsArg4*: F_7, 24_ = 2.212, *P* = 0.0977). Similar to development profiles, reference gene effect was significant for *NlylsSDH* and *EdePRTase* but not for *ylsArg4* (*NlylsSDH*: F_2, 24_ = 4.449, *P* = 0.0319; *EdePRTase*: F_2, 24_ = 4.471, *P* = 0.0315; *ylsArg4*: F_2, 24_ = 1.829, *P* = 0.1969). Significant interaction effect was found for all target genes (*NlylsSDH*: F_14, 24_ = 37.064, *P* < 0.001; *EdePRTase*: F_14, 24_ = 26.201, *P* < 0.001; *ylsArg4*: F_14, 24_ = 27.376, *P* < 0.001) suggesting that the impact of reference genes varied among samples of different growth stages. Figure 6 shows the effect of different reference genes on the target gene expression levels for each developmental stage.

**Figure 7:**
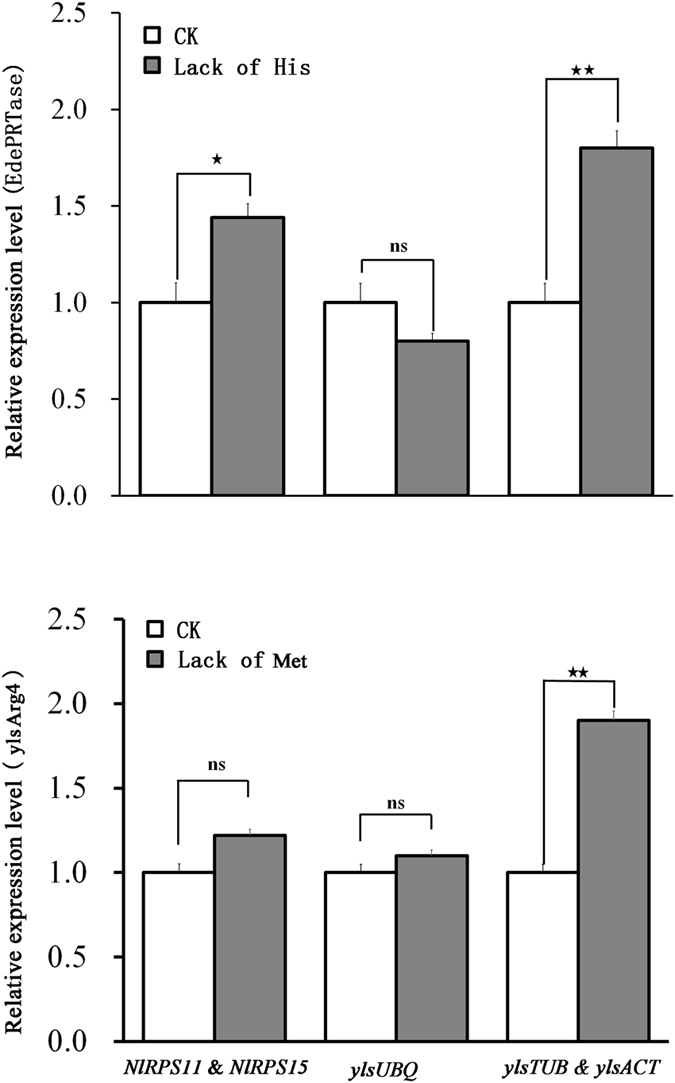
Validation of reference gene selection in samples representing different nutrition diets. The expression of target genes *EdPRTase* (**A**) and *ylsArg4* (**C**) was determined in artificial diet D-97 (CK) and histidine- or methionine-free diet, respectively. The data are denoted as mean ± SE. The pentagram above the vertical bars represent significant differences (*P* = 0.05) (Student’s *t* test). Ns, not significant.

Furthermore, the mRNA responses of *EdePRTase* or *ylsArg4* to histidine- or methionine-free artificial diets were determined as *EdePRTase* is involved in the biosynthesis of histidine and *ylsArg4* is involved in the biosynthesis of methionine. The results showed that differences were present among the tests of the different reference genes (Fig. 7). For *EdePRTase*, both reference gene pairs of *NlRPS11*/*NlRPS15* and *ylsTUB*/*ylsACT* detected the expected difference of expression between normal and histidine deprived diets (Fig. 7A), while the unsuitable reference gene *ylsUBQ* failed. Using *NlRPS11/NlRPS15* as reference genes, the expression levels of *EdePRTase* that responded to the histidine-free artificial diet was consistent with published results^36^. For *ylsArg4*, only *ylsTUB*/*ylsACT* reference gene pair test was able to detect the expected difference between normal and methionine deprived diets (Fig. 7B).

**Figure 8:**
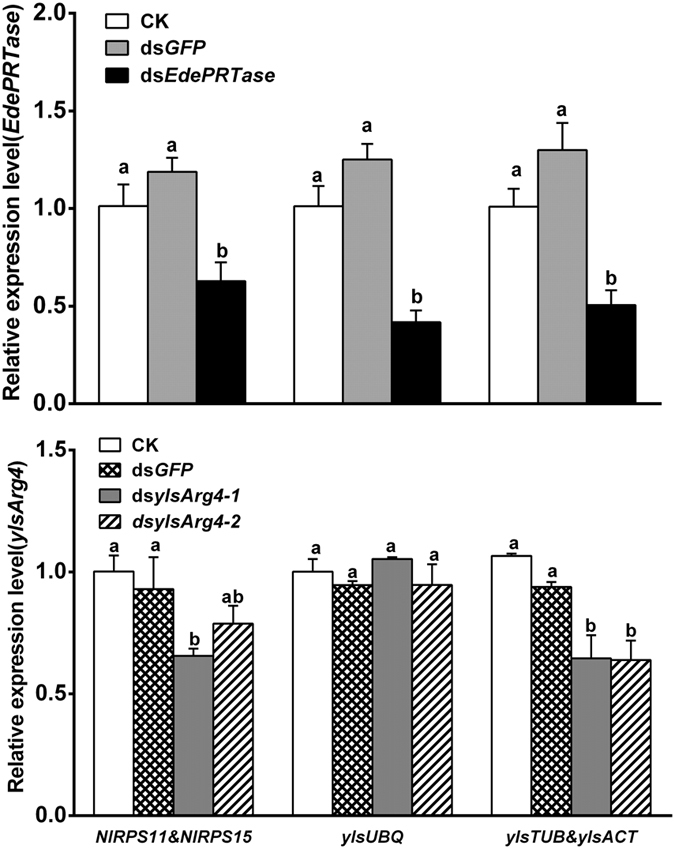
Validation of reference gene selection in samples of dsRNA injection. The expression of target genes *EdPRTase* (**A**) and *ylsArg4* (**B**) was determined in ds*EdePRTase* or ds*ylsArg4* (ds*ylsArg4-1* and ds*ylsArg4-2*) injected samples, respectively. The data are denoted as mean ± SE. Different letters above the vertical bars represent significant differences (*P* = 0.05) (Turkey’s test). Ns, not significant.

All three RNAi samples tested with different reference genes were able to detect *EdePRTase* knockdowns with similar trends (Fig. 8A). However, the knockdown of *ylsArg4-1* was detected by the tests with reference genes of *NlRPS11/NlRPS15* and *ylsTUB*/*ylsACT.* The knockdown of *ylsArg4-2* was only detected by the test with reference genes of *ylsTUB*/*ylsACT.* The deemed unsuitable reference gene *ylsUBQ* failed to detect any knockdowns (Fig. 8B).

## Discussion

Reference gene(s) selection is a crucial component of RT-qPCR that directly impacts the quality of gene expression profiling. A good reference gene should be expressed consistently regardless of tissue type and experimental condition. Reference genes also need tolerance to differences in starting material amount and quality, and to differences in RNA extraction and cDNA synthesis as they are exposed to the same preparation procedure of the target gene(s)^37^,^38^. On the other hand, a gene in living cells cannot be totally immune to changes responding to physiological and nutritional status due to experimental manipulations. It is important to identify the least regulated gene(s) of individual experiment as reference genes for accurate RNA transcription analysis. The current study focused on YLS of *N. lugens* as YLS is crucial for the survival of the hosts^21^. Recent studies have identified multiple YLS genes that play a role in essential amino acid biosynthesis^34^,^36^,^39^, which brings more attention to YLS. Eight YLS genes in *N. lugens* were investigated for their expression stabilities in an effort of identifying suitable reference genes for RT-qPCR studies of holosymbiont (including both *N. lugens* and YLS, *N. lugens*-symbiont YLSs). To our knowledge this is the first report of such kind.

Among the eight evaluated YLS genes, *ylsRPS15e* that encodes one member of ribosomal proteins had the third highest expression level with the penultimate lowest expression variation, comparing to the other genes, suggesting that it would be a good reference gene. A literature search indicates that this is the first report that *RPS15e* is suggested as a reference gene in fungi. In insects, *RPS11* and *RPS15* from *N. lugens* and *ribosomal protein L10* and *ribosomal protein L17A* from *Spodoptera exigua* were proven to be acceptable reference genes, with high expression in all tissue samples^40^,^41^. *GAPDH* is one of the most commonly used reference genes and is often referred as “classical”^42^. The use of *GAPDH* in many studies produced good results, but in some other studies *GAPDH* was not recommended due to expression variability caused by experimental factors^43^. Additionally, GAPDH’s roles outside of the glycolytic pathway that may result in variable expression levels of different tissues^44^,^45^. These results indicate that genes involved in the fundamental processes of cells as candidate reference genes might also be significantly influenced by other processes during experimentation. In the current study, *ylsEF1α* and *ylsGAPDH* showed relatively lower expression levels and higher expression variations, and should be avoided for relative expression analysis in some specific conditions.

For molecular level studies of the interaction between YLS and *N. lugens*, gene expression of different developmental stages are often characterized as the information is important for gene function analysis and the timing of RNAi. Additionally, nutrition and temperature are important experimental factors because of YLS’s role in amino acid biosynthesis and that YLS as fungus are sensitive to temperature manipulation. For these reasons, we evaluated expression stability of the eight YLS genes across developmental stages and identified the best reference gene pair *ylsRPS15p*/*ylsACT*. Similarly, the best reference gene pairs for nutritional and temperature manipulations were identified as *ylsTUB*/*ylsACT*, and *ylsRPS15e*/*ylsGADPH*, respectively. These results should greatly benefit the RT-qPCR studies of *N. lugens* or other organisms that host YLS.

Currently the most YLS studies used genes of its host *N. lugens*, e.g. *RPS11* and *RPS15*, as reference genes regardless of experimental conditions. Our study shows that endogenous reference genes should be selected depending on experimental settings and objectives. To verify our reference gene selections, we conducted comparative studies of the three recommended reference gene systems using the selected target genes. For *NlylsSDH* (encodes saccharopine dehydrogenase that catalyzes the penultimate reaction in the biosynthesis of the amino acid lysine), *ylsRPS15p*/*ylsACT* reference genes had the advantage of detecting higher expression levels in fourth- and fifth-instar nymphs, compared to the reference genes of *ylsUBQ* and *NlRPS11*/*NlRPS15.* Previous study using *NlRPS15* and *NlTUB* as internal references had suggested that *NlylsSDH* had higher expression levels in first to fourth instar nymphs than that in fifth instar nymph and adults^34^. However, when RNAi experiment was carried out for the first instar nymphs, no significant adverse effect on body weights and nymphal development durations were observed, suggesting that the gene may not be highly expressed in the 1^st^ instar nymphs. Performing RNAi during the 4^th^ or 5^th^ instar may yield better results. Target gene *EdePRTase* (also originated from YLS) is ubiquitously but unevenly expressed among the different life stages using the reference genes of *N. lugens*, *NlRPS11* and *NlRPS15* 36. In the current study, *EdePRTase* had the highest expression level in the fourth and fifth instar nymphs using all three reference gene systems, which is partially consistent with the published expression patterns^36^. The *EdePRTase* RNAi experiments were successful, with significant down-regulated expression of *EdePRTase* and histidine level patterns.

More importantly, the current study demonstrated the importance of reference gene selections for different experimental objectives. *EdePRTase* is a gene that contributes to the biosynthesis of histidine. Both YLS and *N. lugens* reference genes were able to detect the expected expression difference due to the methionine deprived diet. However, for *ylsArg4*, an YLS gene involved in the biosynthesis of methionine, only *ylsTUB*/*ylsACT* reference gene pair was able to detect the expected difference between the normal and methionine deprived diets. Similarly, RNAi experiments showed that gene knockdown of *EdePRTase* was detected by all the reference genes used with similar trends, while the knockdown of *ylsArg4-1* was detected by reference genes of *NlRPS11/NlRPS15* and *ylsTUB*/*ylsACT*, and the knockdown of *ylsArg4-2* was only detected by the use of reference genes of *ylsTUB/ylsACT.* Thus, YLS genes seem more tolerant to experimental manipulations. These results clearly demonstrate the advantage of using endogenous YLS genes over exogenous host genes *NlRPS11* and *NlRPS15*.

## Conclusion

Tailored reference gene selection is deemed necessary for gene transcription studies by RT-qPCR. For organisms hosting microbial endosymbionts, using endogenous genes of the symbionts as reference genes may possess advantages over host genes. The current study concludes that *ylsRPS15p* and *ylsACT* are the most suitable reference genes for temporal gene expression profiling, while *ylsTUB* and *ylsACT,* and *ylsRPS15e* and *ylsGADPH* are the most suitable reference gene choices for evaluating nutrition and temperature effects. Such specific reference gene recommendations should benefit future gene expression studies of *N. lugens* and its YLS. To our knowledge, this is the first study that investigates different candidate reference genes for gene expression analyses and seems to be useful in guiding researchers in performing gene expression analyses in this species.

## Materials and Methods

### Ethics statement

All animal work has been conducted according to the relevant national and international guidelines.

### Insect sample preparation

*A N. lugens* colony was maintained on rice (*Oryza sativa*) variety Taichung Native 1 (TN1, a hopper susceptible variety) in a greenhouse of the China National Rice Research Institute under the conditions of 28 ± 1 °C, 80 ± 10% relative humidity and a 16 L/8D photoperiod^34^,^46^. Eggs, first- to fifth-instar nymphs (I1, I2, I3, I4 and I5), and newly emerged adults (female and male) (<24 h after molting) were collected and frozen at −80 °C until RNA extraction. Furthermore, a pooled sample including eggs, nymphs (I1-I5) and adults (male and female) was collected for PCR efficiency testing. For the insect samples of amino acid deprivation, newly formed third-instar nymphs were transferred to the standard artificial diet D-97 or modified artificial diets without one of the 20 amino acids^46^. For each diet type, 250 individuals were prepared in groups of 25 (a total of 10 groups). After four days on the diet, five individuals of each group were collected and pooled as one sample (a total of 50 individuals) and frozen at −80 °C until RNA extraction. This experiment was repeated three times as independent biological replicates.

To evaluate the effect of temperature, 180 newly emerged female adults (<24 h after molting) were placed into 18 glass tubes in a group of 10. Three of the tubes (replications) were exposed under one of the 6 temperature regimes (0 °C, 8 °C, 18 °C, 28 °C, 38 °C, 48 °C) for 2 h in an incubator (DC-3010, Jiangnan Equipment, Hangzhou, China). The nymphs then were recovered at 28 ± 1 °C for 2 h. The surviving individuals were frozen in liquid nitrogen and stored at −80 °C.

RNAi samples were prepared according to a previously reported protocol^36^ as the following: four dsRNAs were synthesized from *EdePRTase* (ds*EdePRTase*), *ylsArg4* (ds*ylsArg4-1* and ds*ylsArg4-2*), and the green fluorescent protein (ds*GFP*) as ds*EdePRTase* and ds*ylsArg4* had previous successes of knocking down the targeted genes evaluated using *N. lugens* reference genes *NlRPS11* and *NlRPS15* 36. A total of 90 fourth-instar nymphs (1-day old) in three groups of 30 individuals each (as three replicates) were injected with the four dsRNAs (in 50 nL) (ds*EdePRTase*, 0.5 μg/μL; ds*ylsArg4*, 1.5 μg/μL; and ds*GFP*, 1.5 μg/μL as negative controls, respectively) at the thorax between the mesocoxa and the hind coxa. Insects without dsRNA injection were used as blank control. Three days after the injection, the surviving individuals were collected and pooled for RT-qPCR analysis using the primers listed in Table 1. The experiment was repeated three times as independent biological replicates.

### RNA extraction and cDNA synthesis

Total RNA was extracted from the collected samples using the Trizol Reagent (Invitrogen, Shanghai, China) and the RNeasy Mini Kit (Qiagen, Valencia, CA) according to the manufacturer’s instructions. The RNA purity was measured with a NanoDrop 1000 spectrophotometer (Thermo Fisher Scientific, Rockford, USA) and the integrity was checked by agarose gel electrophoresis. One microgram total RNA was reverse-transcribed to cDNA by using TransScript One-Step gDNA Removal and cDNA Synthesis SuperMix kit (TransGen Biotech, Beijing, China).

### Reference gene candidates from YLS and cloning

A total of eight housekeeping genes was selected from YLS draft genome sequences (Genbank accession numbers: JRMI00000000.1) as candidates of the most consistently expressed reference gene(s) to be used in RT-qPCR studies. They are *γ-actin* (*ylsACT*), *α-tubulin* (*ylsTUB*), *ribosomal protein S11* (*ylsRPS11*), *ribosomal protein S19A*/*S15e* (*ylsRPS15e*), *ribosomal protein S15P/S13E* (*ylsRPS15p*), *elongation factor 1α* (*ylsEF1α*), *glyceraldehyde-3-phosphate dehydrogenase* (*ylsGAPDH*) and *ubiquitin* (*ylsUBQ*). These gene types have been used widely as reference genes in studies of insects and fungi. Primers were designed based on the sequences selected by the PrimerQuest software (Integrated DNA Technologies, Inc, Coralville, IA, USA) (Table 1). Additionally, BLAST analyses were performed to verify their specificity (Fig. 1). The PCR products of the female adults were sequenced by an ABI 3730 automated sequencer (Applied Biosystems, Foster City, CA, USA) in both directions. The obtained sequences were submitted to GenBank database (AB914563-AB914566).

### RT-qPCR

Prior to the RT-qPCR amplification, a portion of cDNA was used to construct standard curves for PCR condition optimization and calculation of PCR efficiency of the eight selected reference gene candidates. For this purpose, five cDNA quantities (0.2, 2, 10, 50, 200 ng) were tested. PCR amplification efficiencies were calculated using the formula E = (10^[−1/slope]^ − 1) × 100.

The RT-qPCR experiments were performed using the TransStart Top Green qPCR SuperMix- -UDG (TransGen Biotech) according to the manufacturer’s instructions on an ABI 7500 Real Time PCR System. Amplification reaction volume was 20 μL with 3 μL cDNA as template and a final concentration of 200 nM primers. The following thermal cycling condition was used: initial denaturation at 95 °C for 2 min, followed by 40 cycles of 95 °C for 20 s and 60 °C for 1 min. After all reactions, a melting curve analysis from 65 °C to 95 °C was applied to ensure consistency and specificity of the amplified product, and the most adequate annealing temperature was determined and used for PCR reaction. Each primer pair was checked for size specificity of the amplicon using 1.5% agarose gel electrophoresis and ethidium bromide staining. A reaction mix without template (no template control) was used to detect possible reagent contamination. The average of the Ct values was determined using manual quantification settings. The values of Ct for the control wells were excluded from further analysis, as these values were greater than 35 or not detectable.

### Determination of expression stability

Stabilities of the reference gene candidates were evaluated by BestKeeper^38^, geNorm^10^, NormFinder^47^ and the ΔCt method^11^. BestKeeper analysis uses Ct values directly, while geNorm, NormFinder and ΔCt method use transformed Ct values of (1 + E)^−ΔCt^. In addition, RefFinder^48^, a web-based comprehensive platform that integrates the four above mentioned algorithms, was used for the overall ranking of the stability of these reference gene candidates. All data were analyzed according to the perspective instructions of the software used.

### Validation of selected reference genes

Three YLS originated genes that are involved in amino acids biosynthetic pathways including *NlylsSDH* 34, *EdePRTase* 36 and *ylsArg4* (unpublished data) were used as target genes to validate the selected YLS reference gene candidates using the 2^−ΔΔCt^ method. Developmental stage and sex samples, samples of methionine- or histidine-free artificial diets, and RNAi samples were used.

## Additional Information

**How to cite this article**: Wan, P.-J. *et al*. Reference genes for quantitative real-time PCR analysis in symbiont *Entomomyces delphacidicola* of *Nilaparvata lugens* (Stål). *Sci. Rep.* **7**, 42206; doi: 10.1038/srep42206 (2017).

**Publisher's note:** Springer Nature remains neutral with regard to jurisdictional claims in published maps and institutional affiliations.

## Figures and Tables

**Table 1:** Primers used for RT-qPCR analysis and dsRNA synthesis.

Gene	Primer sequence (5′ to 3′)	Amplicon size (bp)	PCR efficiency (%)	R^2^
**RT-qPCR**				
*ylsAct*	CATCCACGTAACCACCTACAA	224	105.4	0.999
	AGGCCAAGATAGATCCACCA			
*ylsEF1α*	ACTACCCAGTGGTCCGAGTC	209	104.1	1.000
	AAGGTCTTGCCAGTGGTCTT			
*ylsGADPH*	GCAAAGTCATTCCCGATCTT	196	103.6	0.996
	GAGACGATTTCTTCATCGCA			
*ylsRPS11*	ACGGAATCCCATTCAAAGAG	167	102.5	0.999
	AGCTTGAGCAGCTACATTGG			
*ylsRPS15e*	AAGCCAAGCCTAACGAGAAG	234	99	0.997
	GTGGAATGAAACGAGACGAA			
*ylsRPS15p*	AGCTCTGAGGTCCAGATTGC	193	103.5	1.000
	TCAAGTGGGTCCATCTTTCA			
*ylsTub*	TTGATTGCTCAGGTCGTCTC	196	115.7	0.995
	GGTCATCTCTTGGACGGAGT			
*ylsUBQ*	CTTTGACGGGAAAGACGATT	156	92.8	0.998
	AATCGCTCAGAGTACGTCCA			
*NlylsSDH*	AATGCCTGCTCGTTGATAAC	185	N.A.	N.A.
	ATGTCTTGCCCTAAGGTGCT			
*ylsArg4*	CGAGTTCGAGCAGATTGAAA	70	N.A.	N.A.
	GATGGCAAATATGCCTGTTG			
*EdePRTase*	CGAATCCAGACATGATCGAC	165	N.A.	N.A.
	AGCCGTCATCCAGTGATGTA			
**dsRNA synthesis**				
*dsEdePRTase*	T7-AAGAATCTACCGATCGCCCT	532	N.A.	N.A.
	T7-GGGATCTAATCAAGACGGCA			
*dsylsArg4-1*	T7-GACATACGCGGCAGCATT	430	N.A.	N.A.
	T7-CGAGGCAGTAGGAAAGAAGC			
*dsylsArg4-2*	T7-TCACCGAGACCCTACAATG	307	N.A.	N.A.
	T7-TCCGACACCGTCTTGACA			
*dsGFP*	T7-CCTGAAGTTCATCTGCACCAC	355	N.A.	N.A.
	T7-TGATGCCGTTCTTCTGCTTGT			

Note: T7 RNA polymerase promoter sequence (5′-TAATACGACTCACTATAGGG-3′). N.A., not applicable.

**Table 2:** Ranking of the candidate reference genes as determined by RefFinder.

Conditions	Rank	Gene	Geomean of ranking values
Development stage and sex	1	*ylsRPS15p*	1.57
	2	*ylsACT*	1.86
	3	*ylsEF1α*	2.45
	4	*ylsTUB*	4.23
	5	*ylsRPS11*	4.76
	6	*ylsGADPH*	5.48
	7	*ylsRPS15e*	5.96
	8	*ylsUBQ*	7.24
Artificial diet	1	*ylsACT*	1.19
	2	*ylsTUB*	1.41
	3	*ylsEF1α*	3.41
	4	*ylsRPS15e*	4.00
	5	*ylsGADPH*	5.44
	6	*ylsRPS11*	5.45
	7	*ylsRPS15p*	6.24
	8	*ylsUBQ*	8.00
Temperature	1	*ylsRPS15e*	1.41
	2	*ylsGADPH*	2.51
	3	*ylsACT*	2.59
	4	*ylsTUB*	3.64
	5	*ylsEF1α*	4.12
	6	*ylsUBQ*	5.42
	7	*ylsRPS15p*	5.66
	8	*ylsRPS11*	8.00
